# Author Correction: A ferritin-based COVID-19 nanoparticle vaccine that elicits robust, durable, broad-spectrum neutralizing antisera in non-human primates

**DOI:** 10.1038/s41467-023-42061-4

**Published:** 2023-10-05

**Authors:** Payton A.-B. Weidenbacher, Mrinmoy Sanyal, Natalia Friedland, Shaogeng Tang, Prabhu S. Arunachalam, Mengyun Hu, Ozan S. Kumru, Mary Kate Morris, Jane Fontenot, Lisa Shirreff, Jonathan Do, Ya-Chen Cheng, Gayathri Vasudevan, Mark B. Feinberg, Francois J. Villinger, Carl Hanson, Sangeeta B. Joshi, David B. Volkin, Bali Pulendran, Peter S. Kim

**Affiliations:** 1 Sarafan ChEM-H, Stanford University, Stanford, CA USA; 2 Department of Chemistry, Stanford University, Stanford, CA USA; 3 Department of Biochemistry, School of Medicine, Stanford University, Stanford, CA USA; 4 Institute for Immunity, Transplantation and Infection, Stanford University School of Medicine, Stanford, CA USA; 5 Vaccine Analytics and Formulation Center, Department of Pharmaceutical Chemistry, University of Kansas, Lawrence, KS USA; 6 California Department of Public Health, Richmond, CA USA; 7 New Iberia Research Center, University of Louisiana at Lafayette, New Iberia, LA USA; 8 IAVI, New York, NY USA; 9 Department of Pathology, Stanford University School of Medicine, Stanford, CA USA; 10 Department of Microbiology and Immunology, Stanford University School of Medicine, Stanford, CA USA; 11 Chan Zuckerberg Biohub, San Francisco, CA 94158 USA

**Keywords:** Protein vaccines, SARS-CoV-2, Antibodies

Correction to: *Nature Communications* 10.1038/s41467-023-37417-9, published online 17 April 2023

The original version of this Article contained an error in the legend of Figure 2, which incorrectly read ‘Neutralization titers against Wuhan-1 SARS-CoV-2 pseudovirus for serum obtained from individual animals 21 days following immunization with DCFHP-alum’. The correct version states ‘42 days’ in place of ’21 days’. This has been corrected in both the PDF and HTML versions of the Article.

