# Supplementary material for: A ferritin-based COVID-19 nanoparticle vaccine that elicits robust, durable, broad-spectrum neutralizing antisera in non-human primates
Source: Nat Commun. 2023 Apr 17;14:2149. doi: 10.1038/s41467-023-37417-9 (PMC10110616; doi:10.1038/s41467-023-37417-9)
Supplement: Supplementary file 1 — Supplementary Information [file 41467_2023_37417_MOESM1_ESM.pdf]

Supplementary Information For

A ferritin-based COVID-19 nanoparticle vaccine that elicits robust, durable, broad-spectrum neutralizing antisera in non-human primates

Payton A.-B. Weidenbacher[1,2,‡], Mrinmoy Sanyal[1,3,‡], Natalia Friedland[1,3,‡], Shaogeng Tang[1,3], Prabhu S. Arunachalam[4], Mengyun Hu[4], Ozan S. Kumru[5], Mary Kate Morris[6], Jane Fontenot[7], Lisa Shirreff[7], Jonathan Do[1,3], Ya-Chen Cheng[1,3], Gayathri Vasudevan[8], Mark B. Feinberg[8], Francois J. Villinger[7], Carl Hanson[6], Sangeeta B. Joshi[5], David B. Volkin[5], Bali Pulendran[4,9,10], Peter S. Kim[1,3,11,*]

[1] Sarafan ChEM-H, Stanford University, Stanford, CA, USA
[2] Department of Chemistry, Stanford University, Stanford, CA, USA
[3] Department of Biochemistry, School of Medicine, Stanford University, Stanford, CA, USA
[4] Institute for Immunity, Transplantation and Infection, Stanford University School of Medicine, Stanford, CA, USA.
[5] Vaccine Analytics and Formulation Center, Department of Pharmaceutical Chemistry, University of Kansas, Lawrence, KS, USA
[6] California Department of Public Health, Richmond, CA, USA
[7] New Iberia Research Center, University of Louisiana at Lafayette, New Iberia, LA, USA
[8] IAVI, New York, NY, USA
[9] Department of Pathology, Stanford University School of Medicine, Stanford, CA, USA.
[10] Department of Microbiology and Immunology, Stanford University School of Medicine, Stanford, CA, USA.
[11] Chan Zuckerberg Biohub, San Francisco, California 94158, United States
[‡] These authors contributed equally, listed in reverse alphabetical order
[*] Corresponding author (kimpeter@stanford.edu)

Supplementary Figures:

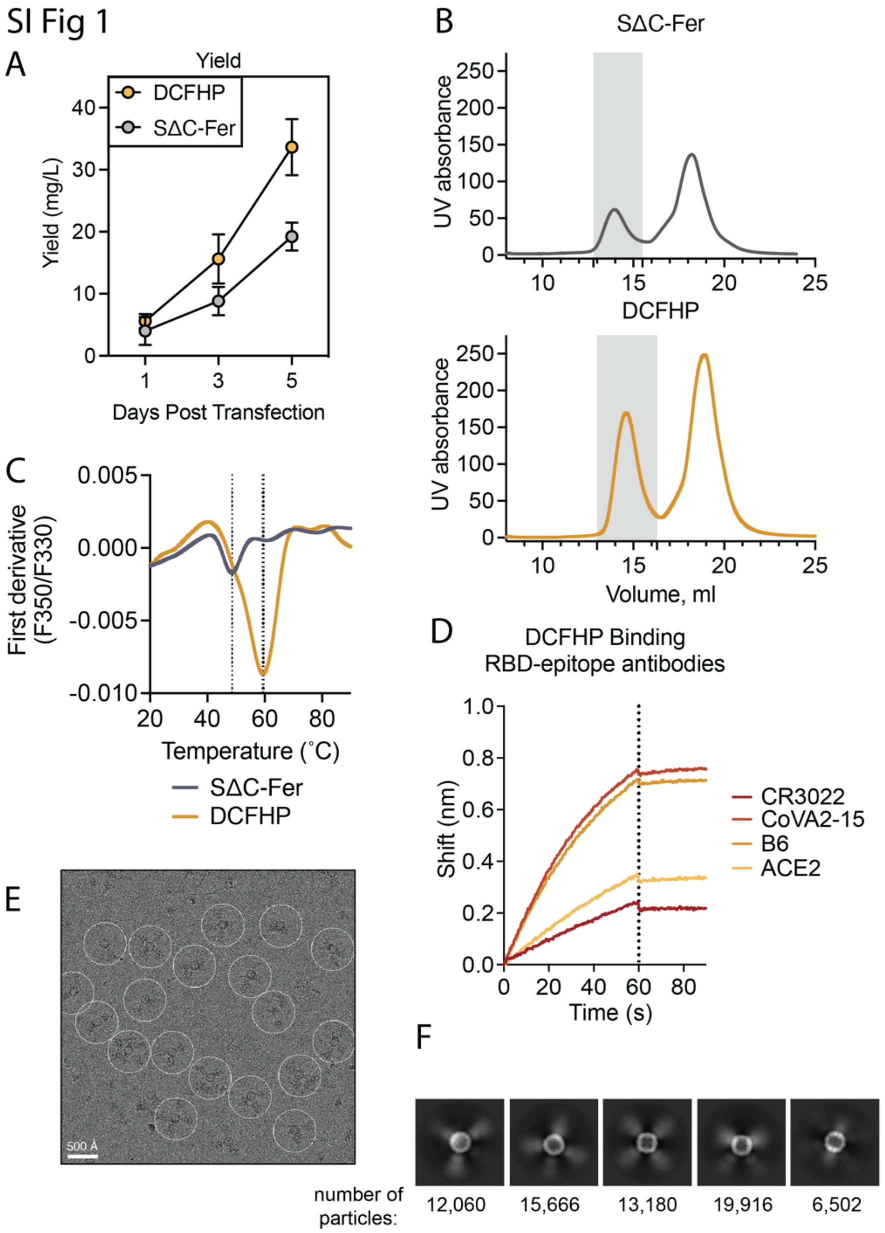

SI Figure 1 – DCFHP shows improved expression compared to SΔC-Fer, as well as proper conformation compared to SΔC-Fer. (A) Yield of DCFHP (yellow) compared to SΔC-Fer (grey) as measured by a normalized BLI assay shows improved expression in an Expi-293F transient transfection model. n=2 replicate experiments, mean and STD are shown. (B) SEC purification on an SRT-1000 column of SΔC-Fer (grey) or DCFHP (yellow) and measurement of UV absorbance at 210 nm on the Akta Pure shows improved nanoparticle yield (grey box indicates pooled fractions). (C) DSF melting profiles of DCFHP (yellow) are substantially altered compared to SΔC-Fer (grey), consistent with previous reports of stabilization conferred by HexaPro mutations (ref[1]). (D) Monitoring binding of antibodies and Fc-ACE2 to DCFHP by BLI indicates proper epitope exposure. (E) Representative motion-corrected Cryo-EM micrograph of DCFHP. White circles indicate single particles that were manually picked and subsequently used as template for particle auto-picking. Scale bar, 500 Å. Image is representative for the of 8,750 movie stacks. (F) Reference-free 2D class averages with the number of particles used in each class. Five 2D classes were used for generating an initial model.

SI Fig 2

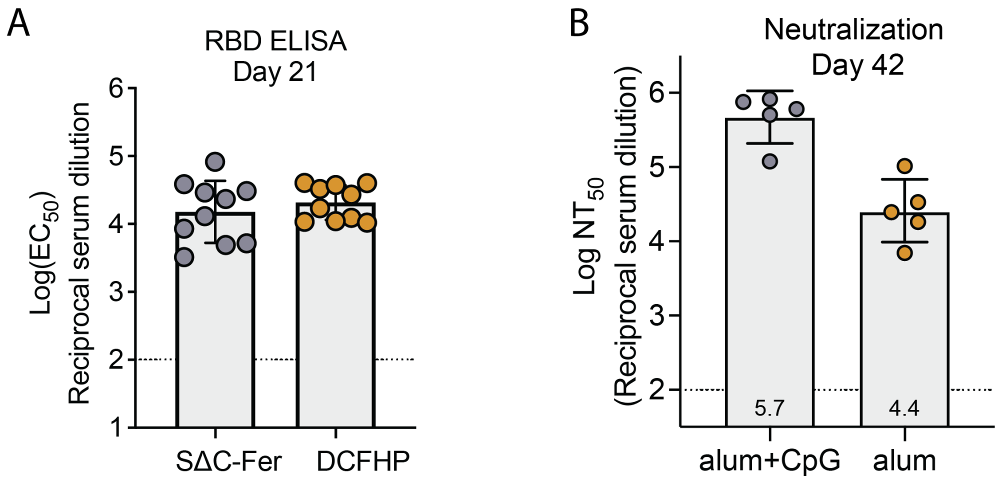

SI Figure 2 – DCFHP is immunogenic and with only alum adjuvant provides robust neutralization. (A) Serum from mice as in Fig 1E tested by ELISA binding to the SARS-CoV-2 RBD by sera from mice isolated at day 21 shows comparable binding between SΔC-Fer and DCFHP. Immunization was done with high-dose alum/CpG. Points are individual titers from each animal, GMT and STD are shown (B) Immunization with DCFHP with alum adjuvant alone retains robust 50% neutralization titer (NT50) against Wuhan-1 SARS-CoV-2 pseudovirus compared to alum and CpG. Serum analyzed on day 42 post a single prime. Individual data points are shown for each animal titer. Assay limit of quantitation are shown as dotted horizontal lines. A single experiment in technical duplicate is shown. GMT and STD are shown.

SI Fig 3

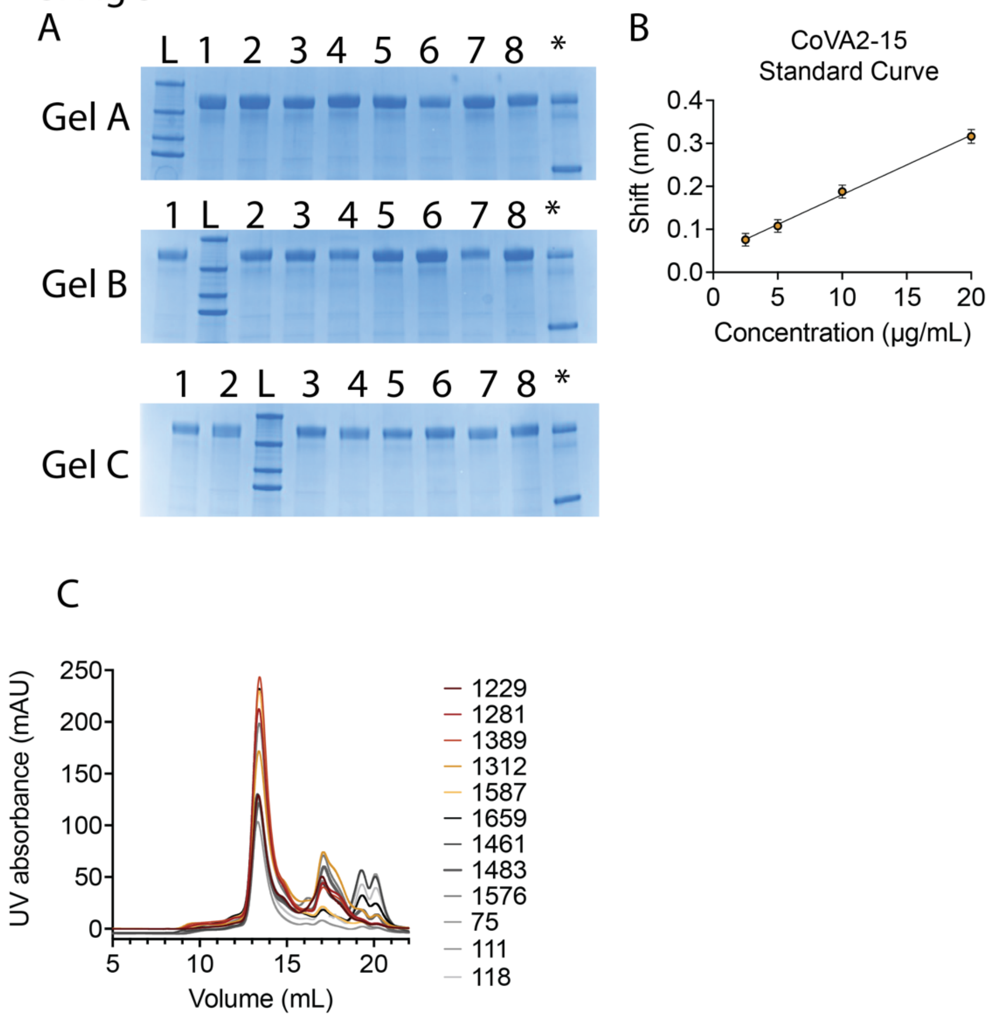

SI Figure 3 – Characterization of 24 lead, single-cell CHO clones expressing DCFHP. (A) SDS-PAGE gels from 0.5µL supernatants of each clone show robust protein expression. L = molecular weight ladder, * = 1µg DCFHP + 1µg BSA. MW lanes are, top to bottom, 250kDa, 150kDa, 100kDa, 75kDa. Gel A lanes 1-8 = C1178, C1229, C1231, C1281, C1312, C1382, C1389, C1396. Gel B lanes 1-8 = C1403, C1461, C1483, C1565, C1576, C1587, C1607, C1659. Gel C lanes 1-8 = C18, C53, C75, C111, C113, C118, C125, C153. (B) The standard curve of DCFHP binding to antibody COVA2-15 shows what was used to convert nm shifts to g/L of clones as described in the Methods. Mean and STD are shown. (C) SEC-MALS traces for 12 samples shown in Gel A, Gel B, and Gel C in panel A define the predominant nanoparticle peak. AUC was used to estimate g/L. as described in the Methods.

SI Fig 4

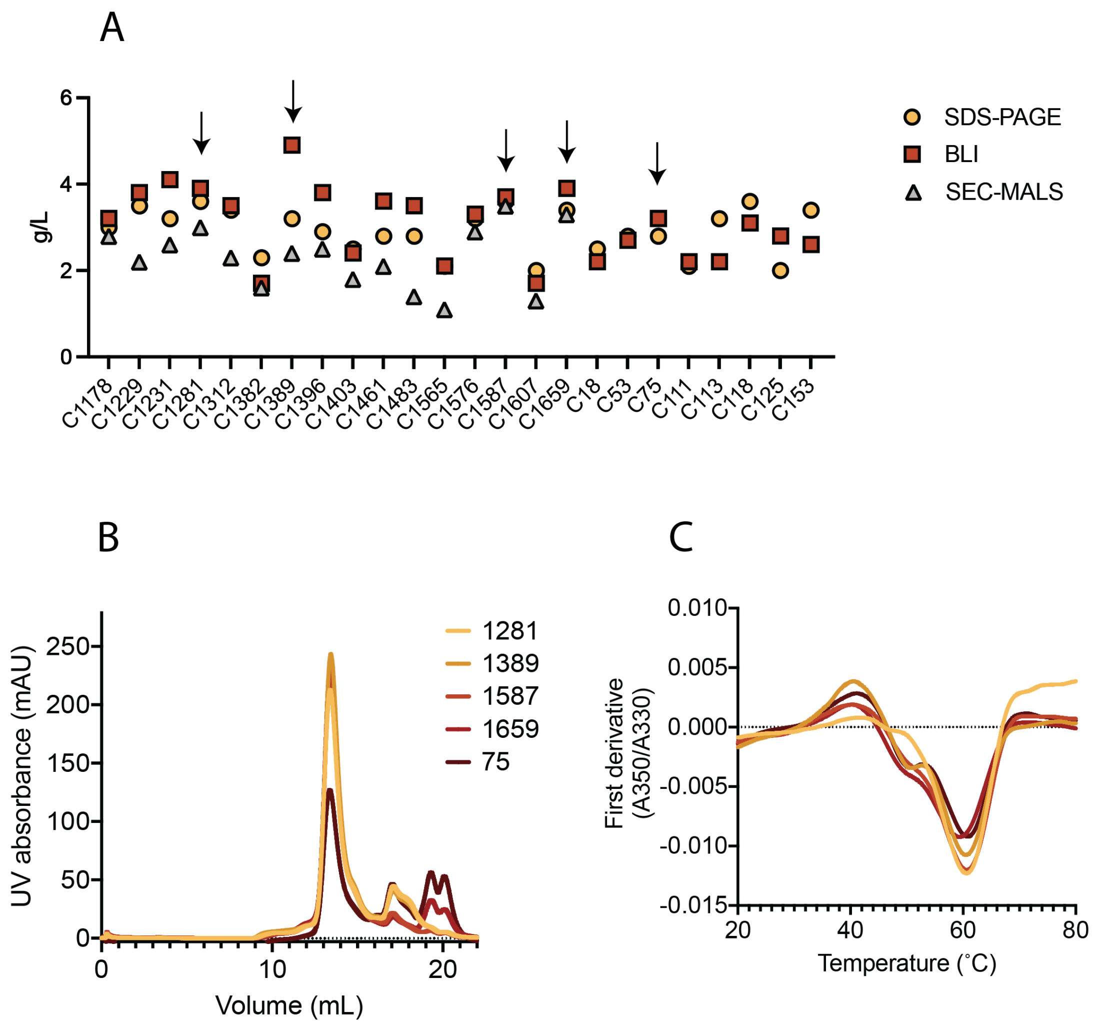

SI Figure 4 – Single-cell clonal selection of DCFHP-expressing, stably integrated CHO K1 cells. (A) The 24 top cell clones were analyzed for DCFHP expression on day 13 of culture by SDS-PAGE, BLI, and SEC-MALS (as in SI Fig 3). Final, calculated g/L are plotted, estimated by densitometry compared to purified DCFHP, a BLI standard curve using CoVA2-15 antibody developed against purified DCFHP, and area under the curve analysis for the nanoparticle peak, respectively. The five clones with the most favorable parameters are indicated by arrows. (B) SEC-MALS traces of the five selected clones shows predominant nanoparticle peaks at ~13.5mL. (C) DSF melting curves of the five selected clones shows similar profiles, with peaks at 40˚C and 61˚C.

SI Fig 5

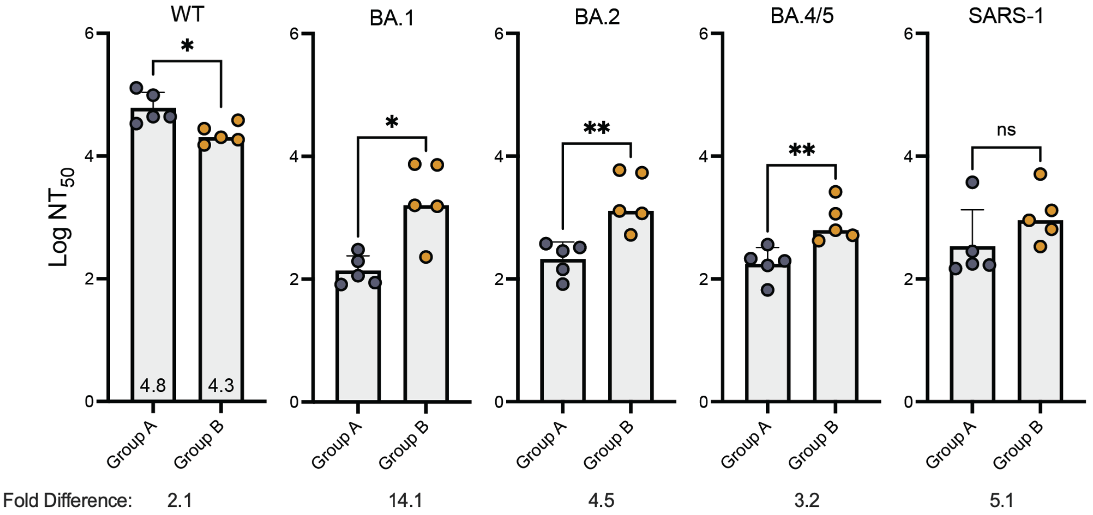

SI Figure 5 – Group A and group B show significant differences in neutralizing potency against Wuhan-1 and Omicron variants 14 days after the first boost. NT50 values were compared between group A and group B, 14 days after the first boost (days 35 and 106 respectively) and significance was tested using a non-parametric, two-tailed Mann-Whitney test. Fold difference between group with highest titer to lower titer group shown on bottom. ns = P > .05, * = P ≤ .05, ** = P ≤ .01, *** = P ≤ .001, **** = P ≤ .0001. Values and replicates are identical to Fig 3, n=4, 4, 1, 3, 1, 2, 4, 5, 2, 5, respectively, defined as in Fig 3. GMT is shown. P = 0.0159, 0.0159, 0.0079, 0.0079, 0.0952, respectively.

SI Fig 6

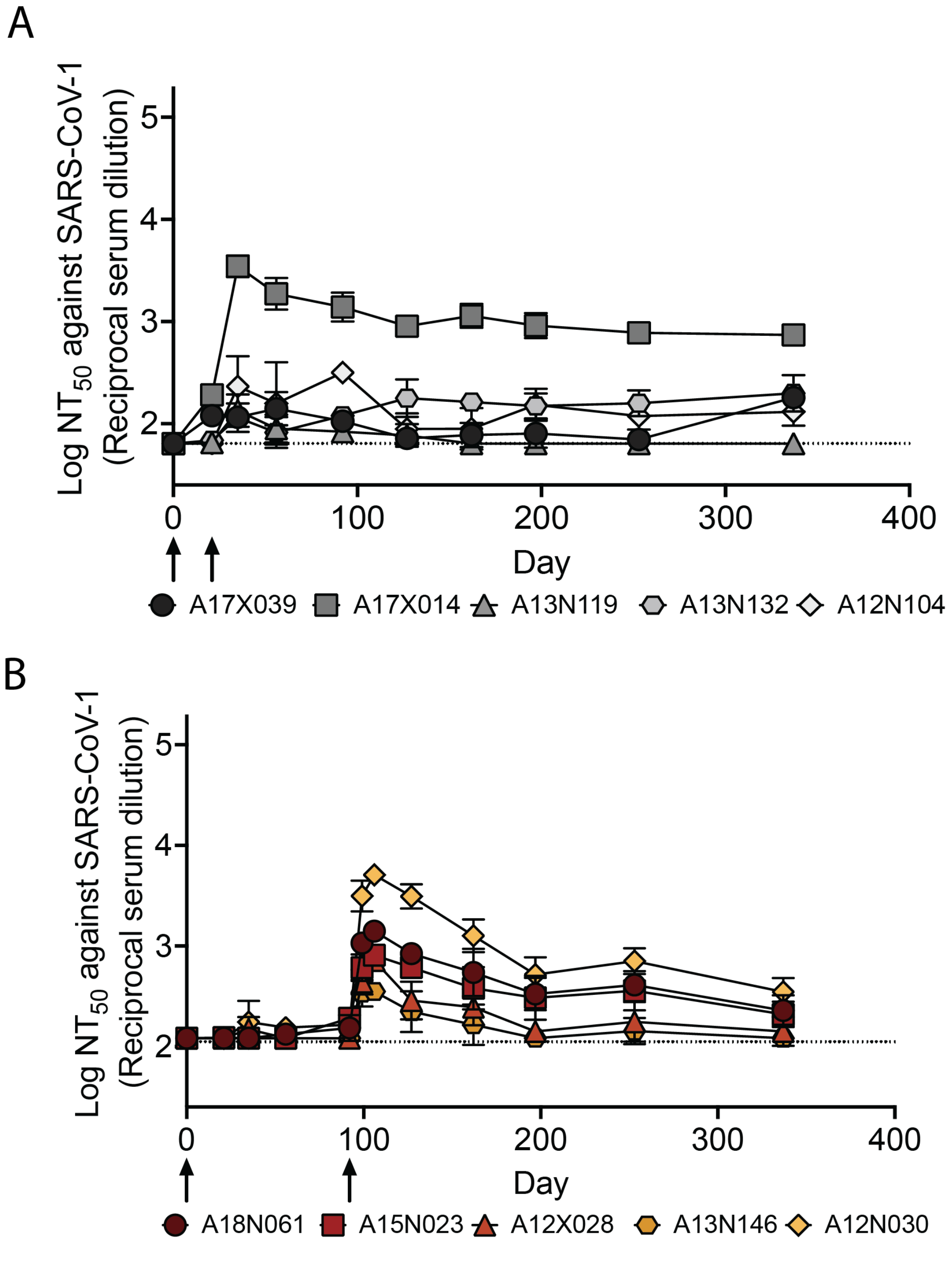

SI Figure 6 – Serum neutralizing titers over 337 days for animals in groups A and B shows longevity of the neutralizing response against SARS-CoV-1 for animals in group B. (A) as in Fig 4A but with SARS-CoV-1 pseudovirus. (B) as in Fig 4C but with SARS-CoV-1 pseudovirus. Average and standard deviation for biological replicates are shown, n = 2 for group A and n = 3 for group B, all biological replicates of the entire experiment were conducted on different days. GMT and STD (for each animal) are shown.

SI Fig 7

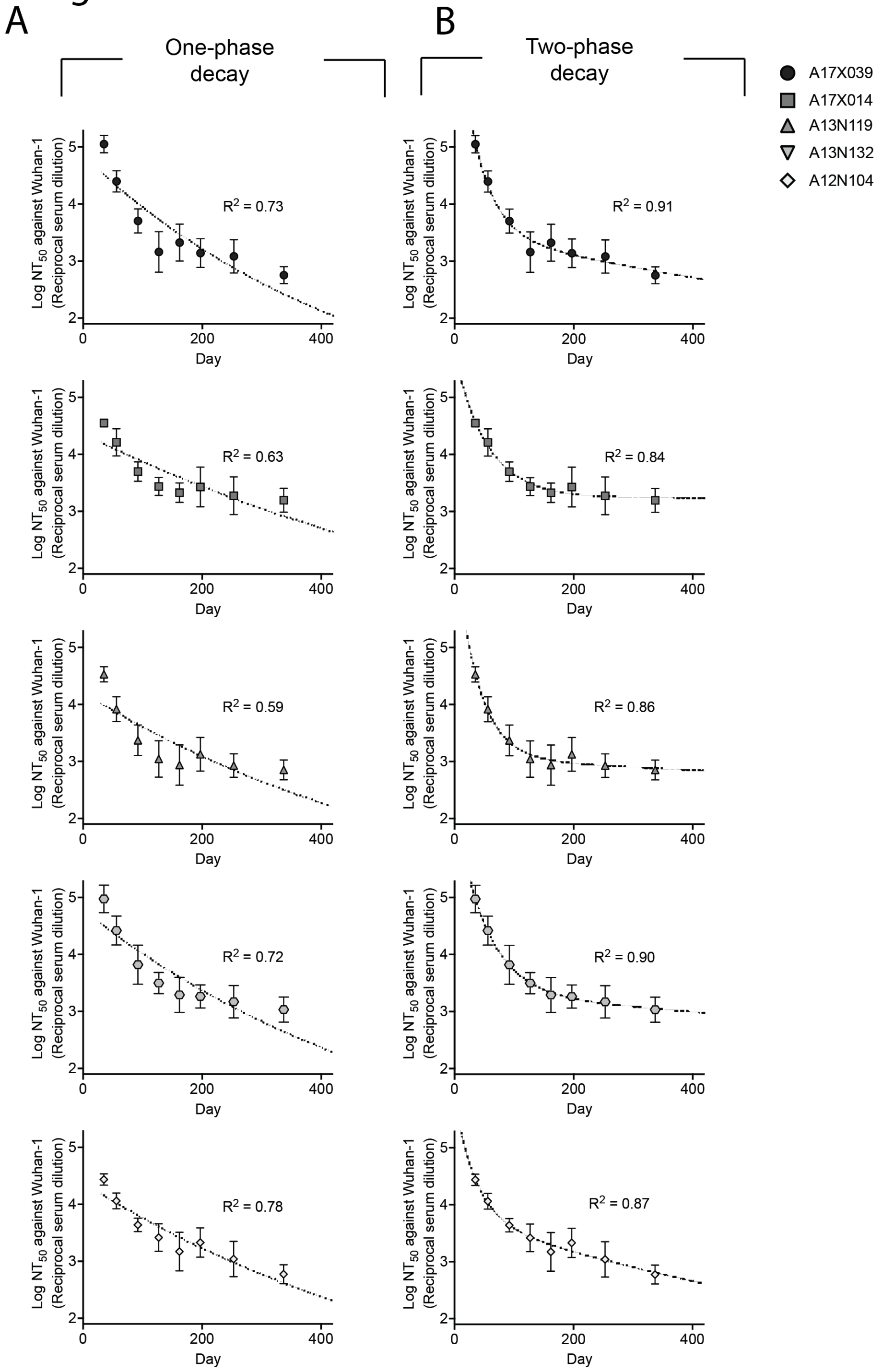

SI Figure 7 – Serum NT_{50} values are best fit with a two-phase decay. Constrained with a plateau value of 0 (NT_{50} value = 1 on log plot), monophasic (A) or biphasic (B) decay models were used to fit the NT_{50} values for individual animal in group A starting 14 days post boost. R^2 values shown on plots. Unconstrained monophasic decay shows acceptable fits, but results in plateau values of ~10^3. Data is the same as in Fig 4A, n=3 throughout.

SI Fig 8

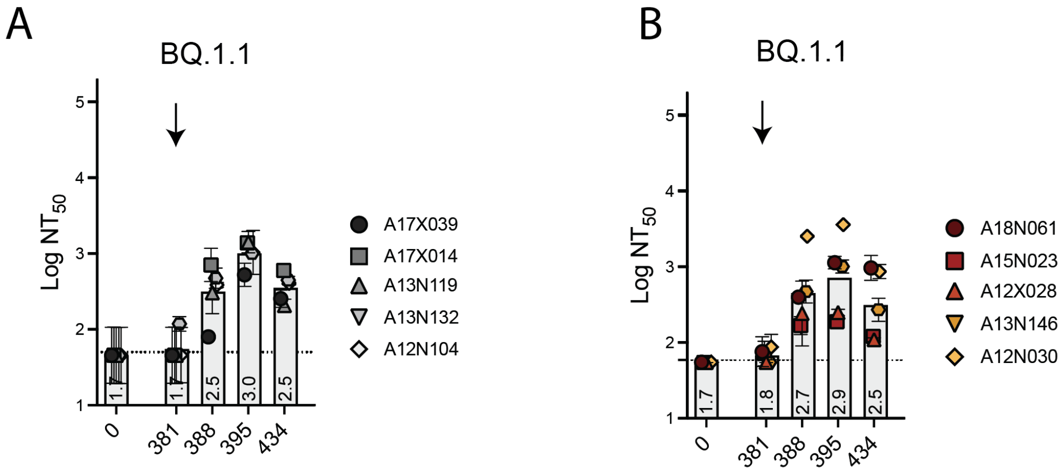

SI Figure 8 – Anamnestic responses against BQ.1.1 is seen following a second booster of DCFHP-alum after ~one year in NHPs. Neutralization against BQ.1.1 by antisera from NHPs in group A (A) or B (B) following a boost at day 381. NHP identification provided correlate with SI table 1. (n = 2 biological replicates throughout). Assay limits of quantitation is indicated by horizontal dotted lines. GMT (bars) and STD (for each animal) are shown.

SI Fig 9

A

Gating for T cells

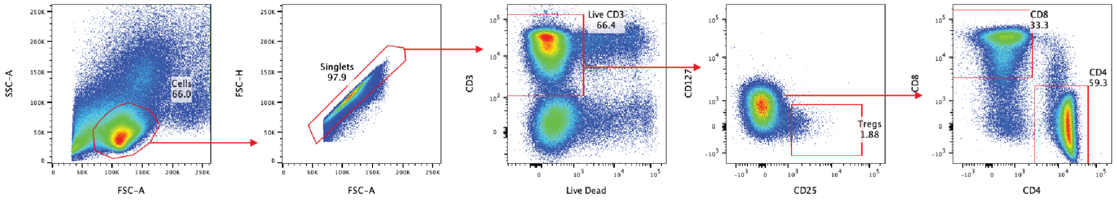

B

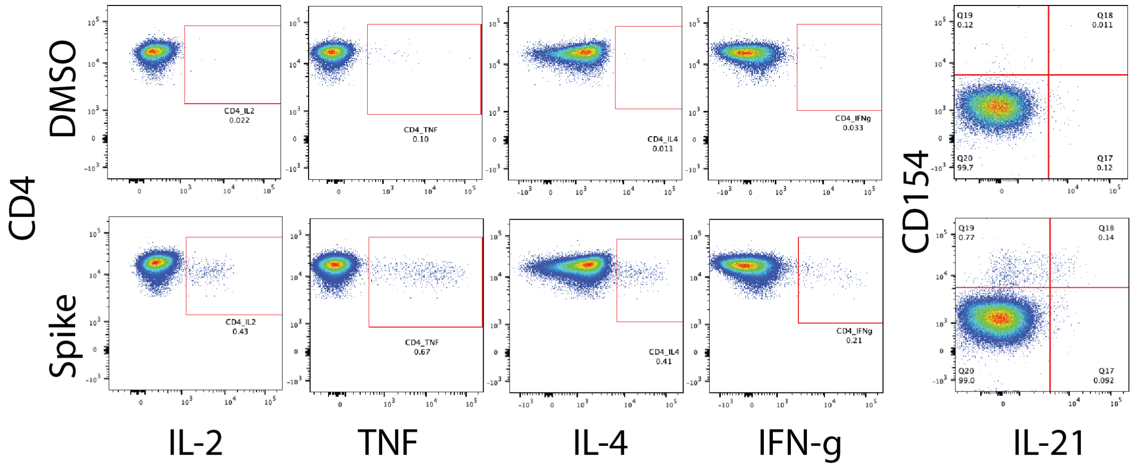

SI Figure 9 – Representative flow cytometry gating scheme for T-cell analysis. (A) Gating scheme for CD4+ T-cells is shown, showing selection of live, single cell T cells which are positive for either CD4+ or CD8+. No CD8+ cells showed stimulation. (B) Representative flow plots from NHP PBMCs stimulated with DMSO (top) or spike protein peptides (bottom), gated as in A were further gated based on intracellular cytokine activity. Stimulation results in positive populations when staining for intracellular cytokines (bottom). Percent positive cells denoted on plots. Percent positive was determined by subtracting the percent positive in the DMSO sample to that in the stimulated sample.

Supplementary Tables:

SI Table 1: NHP Information

Group	Animal ID	Birthdate	Sex	Body Weight (kg) – Day 0 (10/04/2021)
A	A17X039 Rhm	5/16/17	Male	7.5
A	A17X014 Rhm	4/23/17	Male	6.5
A	A13N119 Rhm	6/5/13	Male	8.5
A	A13N132 Rhm	6/19/13	Male	12.5
A	A12N104 Rhm	6/23/12	Male	13.1
B	A18N061 Rhm	4/25/18	Male	5.2
B	A15N023 Rhm	4/9/15	Male	10.3
B	A12X028 Rhm	11/22/12	Male	10.7
B	A13N146 Rhm	6/28/13	Male	12.5
B	A12N030 Rhm	5/1/12	Male	10.8

SI Table 2: NHP Immunizations

Group	Vaccine	Dose (mg)	Adjuvant – Alhydrogel (µg)	Administration Volume (µL)	# of Animals	Immunization Schedule (days)
A	DCFHP	50	750	500	5	0, 21, 381
B	DCFHP	50	750	500	5	0, 92, 381

SI Table 3: Variant of Concern Mutations used in Pseudoviral Assay

Variant	Strain name	Mutations
D614G	D614G	D614G
Alpha	B.1.1.7	Δ69-70, Δ144, N501Y, A570D, D614G, P681H, T716I, S982A, D1118H
Beta	B.1.351	L18F, D80A, D215G, Δ242-244, R246I, K417N, E484K, N501Y, D614G, A701V,
Gamma	P1	L18F, T20N, P26S, D138Y, R190S, K417T, E484K, N501Y, D614G, H655Y, T1027I
Delta	B.1.617.2	T19R, T95I, G142D, Δ156-157, R158G, L452R, T478K, D614G, P681R, D950N
Omicron	BA.1	A67V, Δ69-70, T95I, Δ142-Δ144, Y145D, Δ211, L212I, G339D, S371L, S373P, S375F, K417N, N440K, G446S, S477N, T478K, E484A, Q493R, G496S, Q498R, N501Y, Y505H, T547K, D614G, H655Y, N679K, P681H, N764K, D796Y, N856K, Q954H, N969K, L981F
Omicron	BA.2	T19I, Δ24-26, A27S, G142D, V213G, G339D, S371F, S373P, S375F, T376A, D405N, R408S, K417N, N440K, S477N, T478K, E484A, Q493R, Q498R, N501Y, Y505H, D614G, H655Y, N679K, P681H, N764K, D796Y, Q954H, N969K
Omicron	BA.4/5	T19I, Δ24-26, A27S, G142D, V213G, G339D, S371F, S373P, S375F, T376A, D405N, R408S, K417N, N440K, L452R, S477N, T478K, E484A, F486V, Q493R, Q498R, N501Y, Y505H, D614G, H655Y, N679K, P681H, N764K, D796Y, Q954H, N969K
Omicron	BQ.1	T19I, Δ24-26, A27S. Δ69-70, G142D, Δ144, V213G, G339D, S371F, S373P, S375F, T376A, D405N, R408S, K417N, N440K, K444T, L452R, N460K, S477N, T478K, E484A, F486V, Q498R, N501Y, Y505H, D614G, H655Y, N679K, P681H, N764K, D796Y, Q954H, N969K
Omicron	BQ.1.1	T19I, Δ24-26, A27S. Δ69-70, G142D, V213G, G339D, R346T, S371F, S373P, S375F, T376A, D405N, R408S, K417N, N440K, K444T, L452R, N460K, S477N, T478K, E484A, F486V, Q498R, N501Y, Y505H, D614G, H655Y, N679K, P681H, N764K, D796Y, Q954H, N969K

SI Table 4: Estimated one-phase and two-phase decay half-life values against Wuhan-1 pseudovirus for NHPs

**Animal ID**	Half-Life One-Phase (days)	Half-Life Two Phase, Fast Phase (days)	Percent in Fast Phase (Two Phase)	Half-Life Two Phase, Slow Phase (days)
Group A (Day 21 boost)				
A17X039	340	26	54%	1100
A17X014	580	38	44%	>5000
A13N119	450	26	55%	3,900
A13N132	390	36	50%	2,600
A12N104	450	25	36%	800
Group B (Day 92 boost)				
A18N061	360	47	72%	>5000
A15N023	870	58	48%	>5000
A12X028	200	34	84%	860
A13N146	470	25	84%	1,800
A12N030	380	16	95%	700
Averages for all NHPs	64 weeks ± 25 weeks	4.7 weeks ± 1.8 weeks	47% Group A<br>77% Group B	7.3 years ± 5.1 years

SUPPLEMENTARY REFERENCES

1 Hsieh, C. L. *et al.* Structure-based design of prefusion-stabilized SARS-CoV-2 spikes. *Science* **369**, 1501-1505 (2020). https://doi.org:10.1126/science.abd0826
