# Supplementary material for: A ferritin-based COVID-19 nanoparticle vaccine that elicits robust, durable, broad-spectrum neutralizing antisera in non-human primates
Source: Nat Commun. 2023 Apr 17;14:2149. doi: 10.1038/s41467-023-37417-9 (PMC10110616; doi:10.1038/s41467-023-37417-9)
Supplement: Supplementary file 3 — Reporting Summary [file 41467_2023_37417_MOESM3_ESM.pdf]

nature portfolio

Corresponding author(s): Peter Kim

Last updated by author(s): Feb 28, 2023

Reporting Summary

Nature Portfolio wishes to improve the reproducibility of the work that we publish. This form provides structure for consistency and transparency in reporting. For further information on Nature Portfolio policies, see our Editorial Policies and the Editorial Policy Checklist.

Statistics

For all statistical analyses, confirm that the following items are present in the figure legend, table legend, main text, or Methods section.

n/a	Confirmed	
☐	☒	The exact sample size (n) for each experimental group/condition, given as a discrete number and unit of measurement
☐	☒	A statement on whether measurements were taken from distinct samples or whether the same sample was measured repeatedly
☐	☒	The statistical test(s) used AND whether they are one- or two-sided<br>*Only common tests should be described solely by name; describe more complex techniques in the Methods section.*
☒	☐	A description of all covariates tested
☐	☒	A description of any assumptions or corrections, such as tests of normality and adjustment for multiple comparisons
☐	☒	A full description of the statistical parameters including central tendency (e.g. means) or other basic estimates (e.g. regression coefficient) AND variation (e.g. standard deviation) or associated estimates of uncertainty (e.g. confidence intervals)
☐	☒	For null hypothesis testing, the test statistic (e.g. F, t, r) with confidence intervals, effect sizes, degrees of freedom and P value noted<br>*Give P values as exact values whenever suitable.*
☒	☐	For Bayesian analysis, information on the choice of priors and Markov chain Monte Carlo settings
☒	☐	For hierarchical and complex designs, identification of the appropriate level for tests and full reporting of outcomes
☒	☐	Estimates of effect sizes (e.g. Cohen's d, Pearson's r), indicating how they were calculated

Our web collection on statistics for biologists contains articles on many of the points above.

Software and code

Policy information about availability of computer code

Data collection	For data collection, we used the following commercial and public software applications: Octet System Data Analaysis Software version 9.0.0.15 for biolayer interferometry data acquisition, Gen5 2.09 for neutralization luminesence data acquisition on ELx 405 BioTek reader. Flow cytometry data were collected using a BD FACS Diva v.8.01 software associated with BD FACS Symphony, Nanotemper Prometheus was used for DSF collection, and an agilent Infinity II HPLC paired with a Wyatt miniDAWN TREOS II MALS detector was used for SEC-MALS collection, for Cryo-EM Titan Krios cryo-electronmicroscope, TEM Beta was used at Stanford-SLAC Cryo-EM CenterS2C2, Micrographs were recorded with EPU with a Gatan K2 Summit direct electron detector.
Data analysis	FlowJo software v 10.0 (Treestar Inc), Adobe illustrator V26.5, ASTRA 7.3.2, Prism 9 (version 9.3.1), cryoSPARC 3.2, UCSF Chimera.

For manuscripts utilizing custom algorithms or software that are central to the research but not yet described in published literature, software must be made available to editors and reviewers. We strongly encourage code deposition in a community repository (e.g. GitHub). See the Nature Portfolio guidelines for submitting code & software for further information.

Data

Policy information about availability of data

All manuscripts must include a data availability statement. This statement should provide the following information, where applicable:

- Accession codes, unique identifiers, or web links for publicly available datasets
- A description of any restrictions on data availability
- For clinical datasets or third party data, please ensure that the statement adheres to our policy

Sequences are described or outlined in SI tables and raw data are plotted as shown or included as tables. Raw data for the figures is included in the source data document. SARS-CoV-2 spike protein sequences were taken from GISAID as outlined in SI table 3.

Human research participants

Policy information about studies involving human research participants and Sex and Gender in Research.

Reporting on sex and gender	N/A
Population characteristics	N/A
Recruitment	N/A
Ethics oversight	N/A

Note that full information on the approval of the study protocol must also be provided in the manuscript.

Field-specific reporting

Please select the one below that is the best fit for your research. If you are not sure, read the appropriate sections before making your selection.

☒ Life sciences ☐ Behavioural & social sciences ☐ Ecological, evolutionary & environmental sciences

For a reference copy of the document with all sections, see nature.com/documents/nr-reporting-summary-flat.pdf

Life sciences study design

All studies must disclose on these points even when the disclosure is negative.

Sample size	Sample size was determined by ethical applications and availability. Sample size is shown in the raw data for the figures.
Data exclusions	No data was excluded from the reported analyses.
Replication	Replication is described in the legends where applicable.
Randomization	Randomization of samples was not necessary as all samples were tested by multiple independent researchers in numerical order.
Blinding	Animal numbers were provided to investigators and therefore not blinded.

Reporting for specific materials, systems and methods

We require information from authors about some types of materials, experimental systems and methods used in many studies. Here, indicate whether each material, system or method listed is relevant to your study. If you are not sure if a list item applies to your research, read the appropriate section before selecting a response.

Materials & experimental systems

n/a	Involved in the study
☐	☒ Antibodies
☐	☒ Eukaryotic cell lines
☒	☐ Palaeontology and archaeology
☐	☒ Animals and other organisms
☒	☐ Clinical data
☒	☐ Dual use research of concern

Methods

n/a	Involved in the study
☒	☐ ChIP-seq
☐	☒ Flow cytometry
☒	☐ MRI-based neuroimaging

Antibodies

Antibodies used	Antibody wild type sequences for the variable regions were identified from literature and cloned accordingly into the Vh and Vl portions of an IgG1 plasmid as defined in the methods. The antibody names and citations are provided. For Western Blots, rabbit anti-human IgG H&L HRP (abcam ab6759), For flow antibodies, anti-CD28 (clone CD28.2, BD Biosciences), anti-CD49d (clone 9F10, BD Biosciences), anti-CXCR3 (BD Biosciences), anti-CXCR5 (BD Biosciences). The remainder of the panel is described in the cited literature (PMID 35288714 DOI: 10.1038/s41590-022-01163-9).
Validation	Custom made antibodies were sequence confirmed following cloning and their binding was validated using known binding partners by biolayer interferometry (BLI). Flow antibodies have been used repeatedly in cited studies and validation has been provided on the manufacturers websites per (PMID 35288714 DOI: 10.1038/s41590-022-01163-9).

Eukaryotic cell lines

Policy information about cell lines and Sex and Gender in Research

Cell line source(s)	Expi293F cells were obtained from ThermoFisher (Cat. num. A14527), HeLa-ACE2-TMPRSS2 cells were obtained from the Jesse Bloom Laboratory (Fred Butch). The Vero E6-TMPRSS2-TSA-ACE2 cells are from BEI obtained from Raul Andino's lab, they are regularly tested (quartely) for mycoplasma. The CHO cell line was produced in collaboration with AUTM bio and have been rigorously screened and validated. HEK293T CLR-3216 have been used for viral production in numerous previous studies.
Authentication	None of the cell lines were authenticated for this study.
Mycoplasma contamination	Cell lines used have not tested positive for mycoplasma contamination.
Commonly misidentified lines (See ICLAC register)	No commonly misidentified cell lines were used in this study.

Animals and other research organisms

Policy information about studies involving animals; ARRIVE guidelines recommended for reporting animal research, and Sex and Gender in Research

Laboratory animals	For all NHPs data on age at study start is described in SI table 1, aged A17X039 - 4, A17X014 – 4, A13N119 – 8, A13N132 – 8, A12N104 – 9, A18N061 – 3, A15N023 – 6, A12X028 – 8, A13N146 – 8, A12N030 – 9, years old at study start. Balb/c female mice (6–8 weeks old) were purchased from The Jackson Laboratory and were maintained at Stanford University with 12 hour light/dark cycles.
Wild animals	No wild animals were used in this study.
Reporting on sex	For all NHPs data on sex is described in SI table 1. Mice used in the studies described were female.
Field-collected samples	No field collected samples were used in this study.
Ethics oversight	NHPs were housed and maintained at the New Iberia Research Center (NIRC) of the University of Louisiana at Lafayette in accordance with the rules and regulations of the Committee on the Care and Use of Laboratory Animal Resources. The entire study (IACUC approval number: 2021-012-8738) was reviewed and approved by the University of Louisiana at Lafayette Institutional Animal Care and Use Committee (IACUC) and Stanford University APLAC committee (Protocol # 34139). Mice were maintained at Stanford University according to the Public Health Service Policy for "Humane Care and Use of Laboratory Animals" following a protocol approved by Stanford University Administrative Panel on Laboratory Animal Care (APLAC-33709).

Note that full information on the approval of the study protocol must also be provided in the manuscript.

Flow Cytometry

Plots

Confirm that:

- [x] The axis labels state the marker and fluorochrome used (e.g. CD4-FITC).
- [x] The axis scales are clearly visible. Include numbers along axes only for bottom left plot of group (a 'group' is an analysis of identical markers).
- [x] All plots are contour plots with outliers or pseudocolor plots.
- [x] A numerical value for number of cells or percentage (with statistics) is provided.

Methodology

Sample preparation	Described in detail in the methods – cells were stained in the presence of 1 µg/mL of anti-CD28 (clone CD28.2, BD Biosciences) and anti-CD49d (clone 9F10, BD Biosciences)
Instrument	BD Symphony Flow Cytometer
Software	BD FACS Diva software. All flow cytometry data were analysed using Flowjo software v10 (TreeStar Inc.).
Cell population abundance	T cells were gated such that 66% of events were cells, 97.9% were singlets, 66.4% were live CD3+, 98.1% were not Tregs, 33.3% of the resultant were CD8+ and 59.3% of the resultant were CD4+.
Gating strategy	Cells were gated for cells, singlets, live/dead, CD3+, CD25-, CD8 or CD4 positive, which were further gated to select Th1 or Th2.

- [x] Tick this box to confirm that a figure exemplifying the gating strategy is provided in the Supplementary Information.
